# What is beyond *Salmonella* gastroenteritis? A case of acute pancreatitis complicating *Salmonella* infection in a child: a case report and literature review

**DOI:** 10.1186/s12887-021-02814-w

**Published:** 2021-08-17

**Authors:** Salwa Al Kaabi, Aysha Al Kaabi, Hasa Al Nuaimi

**Affiliations:** 1 Pediatrics Department, General Pediatrics Division, Tawam Hospital, P.O. Box 15258, Al Ain, UAE; 2 Department of Academic Affairs, Tawam Hospital, Al Ain, United Arab Emirates

**Keywords:** *Salmonella* gastroenteritis, Pancreatitis, Amylase, Lipase

## Abstract

**Background:**

*Salmonella* infection presents itself in a wide variety of ways, ranging from mild self-limited illness to severe systemic disease with multiorgan involvement. Acute pancreatitis (AP) is a very rare complication that is associated with *Salmonella* infection, especially among the pediatric population.

**Case presentation:**

A five-year-old boy presented with a two-day fever and experienced vomiting, diarrhea, and abdominal pain. The boy was admitted as a case of acute gastroenteritis, and *Salmonella* was found in his stool culture. The severity of his abdominal pain during his hospital stay indicated the possibility of AP. A clinical examination and blood workup were performed and showed significant elevation in amylase and lipase, which confirmed the diagnosis of AP.

**Conclusion:**

Although abdominal pain is a common presentation of *Salmonella* infection, the possibility of AP must be considered when the pain is severe and the characteristics of the pain are suggestive of AP. Herein, we report a case of AP complicating *Salmonella* infection in an immunocompetent child.

## Background

Acute pancreatitis (AP) is the most common pancreatic disorder in children, and its incidence is increasing worldwide [1]. Recent studies have estimated the incidence of AP in children to be approximately 1/10,000 children per year, an incidence approaching that in adult patients [2] [3].

At present, there are no evidence-based diagnostic guidelines for the diagnosis of AP in children. The expert definition of pediatric AP from the International Study Group of Pediatric Pancreatitis (INSPPIRE) is modeled after the Atlanta classification in adult patients [4]. As per INSPPIRE criteria, a diagnosis of acute pancreatitis requires at least two of the following [5] [6]:

1. Abdominal pain compatible with acute pancreatitis
2. Serum amylase and/or lipase ≥3 times upper limits of normal
3. Imaging findings consistent with acute pancreatitis

In children, blunt abdominal injuries, multisystem disease, biliary stones or microlithiasis, and drug toxicity are the most common contributing factors of AP [1]. The severity of AP is highly variable, with most patients recovering spontaneously with supportive treatment only. Further, it is not uncommon for AP to lead to death if left untreated [4].

AP complicating *Salmonella* gastroenteritis is a rare complication among the pediatric population. This complication has been reported in adult patients with typhoid fever and nontyphoid *Salmonella* infection, but there are few reported cases in the pediatric population [7]. Herein, we report a case of AP complicating *Salmonella* gastroenteritis infection in an immunocompetent child.

## Case presentation

A five-year-old boy, previously healthy, presented with a two-day history of fever and experienced vomiting, diarrhea, and abdominal pain. Although his fever was high, it was not associated with chills or rigors. Vomiting was frequent but not projectile, nor was the vomit bilious or mixed with blood. The diarrheal stool was watery in nature and mixed with mucus, but no blood was detected. The abdominal pain was severe and colicky in nature, located in the epigastric area with no radiation. The pain was relieved by passing stool, with no other pain-relieving factors, including pain medications. Further, the pain was aggravated by food intake. No abdominal distension, no history of drug ingestion and no recent travel.

A physical examination on admission revealed a highly febrile child with a fever reaching 40 °C, tachycardia and otherwise normal vital signs. He also showed signs that indicated moderate dehydration. His physical examination, including an abdominal examination was unremarkable.

Blood investigations revealed normal complete blood count, with normal renal function and electrolytes. Blood and urine cultures were also normal. He had low bicarbonate level of 14 mmol/L (Normal Range: 22–26 mmol/L) and the rapid antigen detection test was positive for Group A streptococcal (GAS) infection. Table 1.

The patient was admitted with the suspicion of GAS infection (tonsillitis), and his abdominal pain was attributed to mesenteric adenitis, which is usually associated with GAS infection. The patient was started on intravenous (IV) hydration and penicillin G sodium. Further, the patient did not have a sore throat at that stage, and his clinical examination did not reveal any sign of GAS tonsillitis.

On the **second and third days of his hospital stay**, the patient continued to have a high-grade fever, abdominal pain, and diarrhea, but his pain was controlled with IV paracetamol.

On the **fourth day of his hospital stay**, the patient’s abdominal pain worsened, and he complained of severe abdominal pain localized to the epigastric area. This pain was aggravated by food intake and relieved by IV paracetamol and nonsteroidal anti-inflammatory drugs. He continuously spiked a high-grade fever with worsening diarrhea that was associated with tenesmus.

**Fig. 1:**
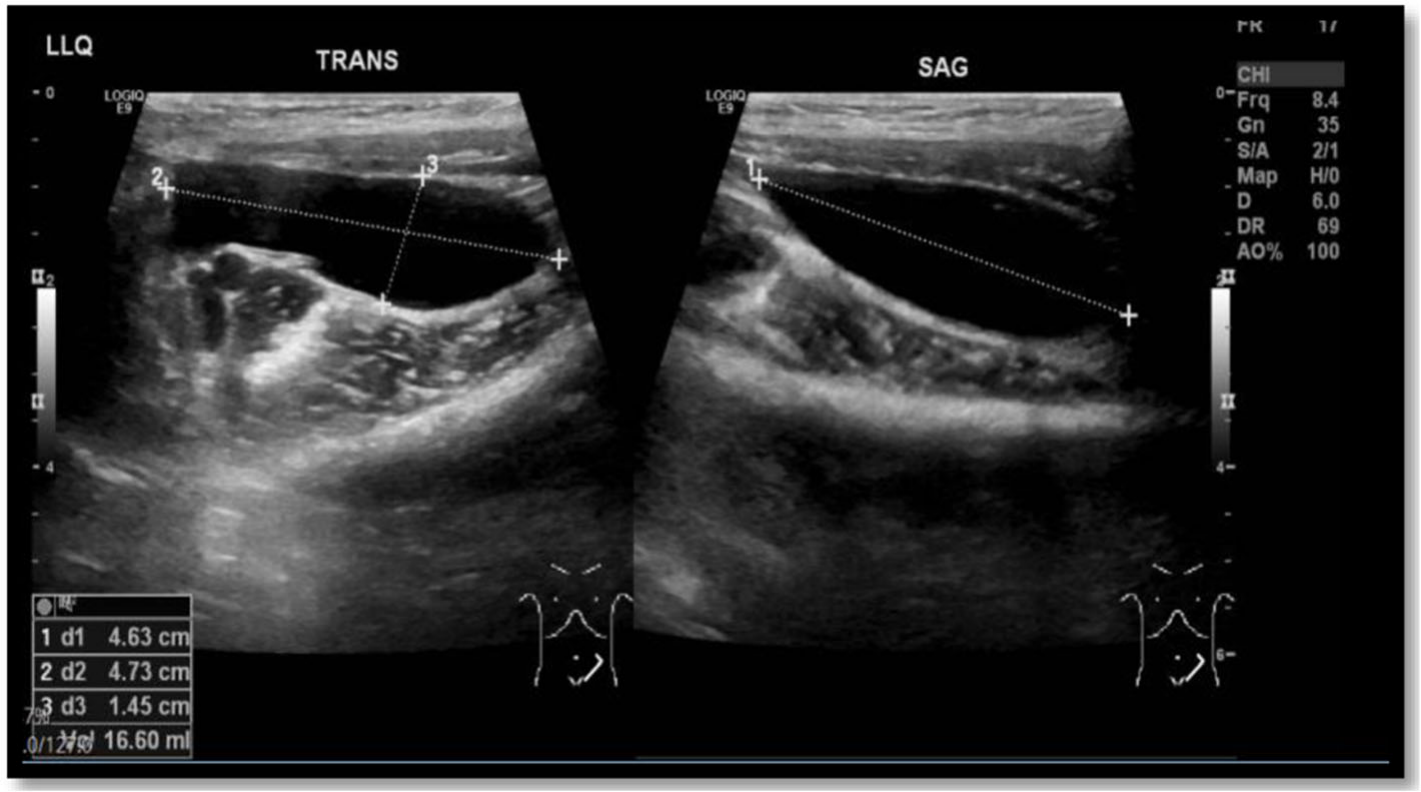
Abdominal US showing a small amount of free fluid (20 ml) in the lower abdomen (mainly in the left lower quadrant)

The examination revealed that the child was highly febrile (temperature above 39 °C) and tachycardic with normal blood pressure. His abdominal examination revealed severe tenderness throughout the abdomen, but a detailed examination could not be performed, as the patient was in severe pain and refused to be examined. The possibilities of an intra-abdominal abscess, acute pancreatitis, surgical abdomen, and bacterial colitis were considered at that stage, and further investigations were conducted to identify any serious pathology. His WBC count remained normal for his age, with a count of 6000/ml (4500–13,500/ml) that mainly showed neutrophil predominance (62%) and immature cells (4%). His hemoglobin dropped to 9.9 g/dL (11.5 g/dL), and his platelet count remained normal. His C-reactive protein (CRP) concentration was checked and was significantly elevated, with a value of 120.6 mg/L (Normal Range: 0–5 mg/L) [8]. His renal and liver function parameters remained normal, and his bicarbonate level improved from 14 mmol/L to 19 mmol/L (22–26 mmol/L). Pancreatic enzymes were elevated, with an amylase level of 101 units/L (Normal Range: 25–101 units/L) and a lipase level of 169 IU/L (Normal Range: 3–32 IU/L) [8]. An abdominal ultrasound (US) was performed to rule out intra-abdominal pathology and showed a small amount of free fluid in the lower abdomen (mainly in the left lower quadrant), with an estimated volume of approximately 20 mL and few prominent mesenteric lymph nodes (Fig. 1). The pancreas was visualized and appeared normal. Mild pelviectasis was also detected, with a diameter of 1 cm and a mildly prominent proximal left ureter.

At this stage, the patient was treated as a case of AP following the standard protocol of nil per os and IV analgesia to control his pain. The possibilities of an intra-abdominal abscess and surgical abdomen could not be ruled out; therefore, the antibiotic was upgraded from penicillin to piperacillin-tazobactam to provide wider coverage for gram-negative enteric organisms and anaerobes. A surgical consultation was obtained, and the surgical team chose supportive management.

**Fig. 2:**
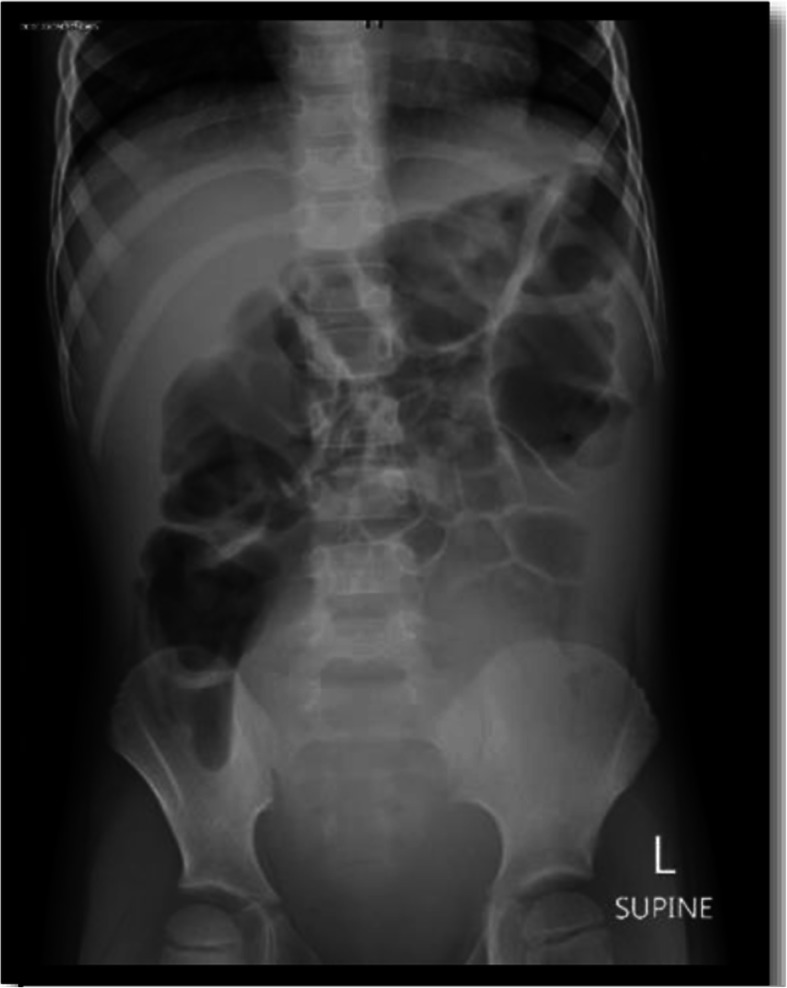
Abdominal Xray showing gas distension of the transverse colon

On **the sixth day of his hospital stay**, the patient still experienced severe abdominal pain; therefore, the abdominal US was repeated, together with an abdominal X-ray. He was scheduled for computed tomography of the abdomen on fourth day of admission to rule out any serious pathology that could have been missed by the US, but the family refused the procedure. Abdominal US showed a fluid pocket of 11 cc in the left iliac fossa (which is an improving compared to the previous scan) and another 8 cc pocket of fluid on the right side, with small para-aortic and mesenteric lymph nodes that were most likely reactive as per the radiologist report. The abdominal X-ray showed gas distension of the transverse colon, with no other abnormalities (Fig. 2). Additionally, pancreatic enzyme analysis was repeated at this stage to check the trend, and upward trends in both enzymes were indicated (amylase increased from 101 units/L to 131 units/L (25–101 units/L) and lipase increased from 169 IU/L to 293 IU/L (3–32 IU/L)). A test of CRP levels was also repeated and showed an upward trend, from 120 mg/L to 123 mg/L (0–5 mg/L). Blood and urine cultures were negative at this stage.

The patient’s condition continued to be managed as a case of pancreatitis. The gastroenterology team’s input was requested at this stage, and they recommended continuing supportive management, monitoring the trend in amylase/lipase on a daily basis, and considering starting a free fluid followed by a fat-free diet. The patient was started on IV morphine for better pain management and omeprazole to avoid stress-induced ulcers.

**Fig. 3:**
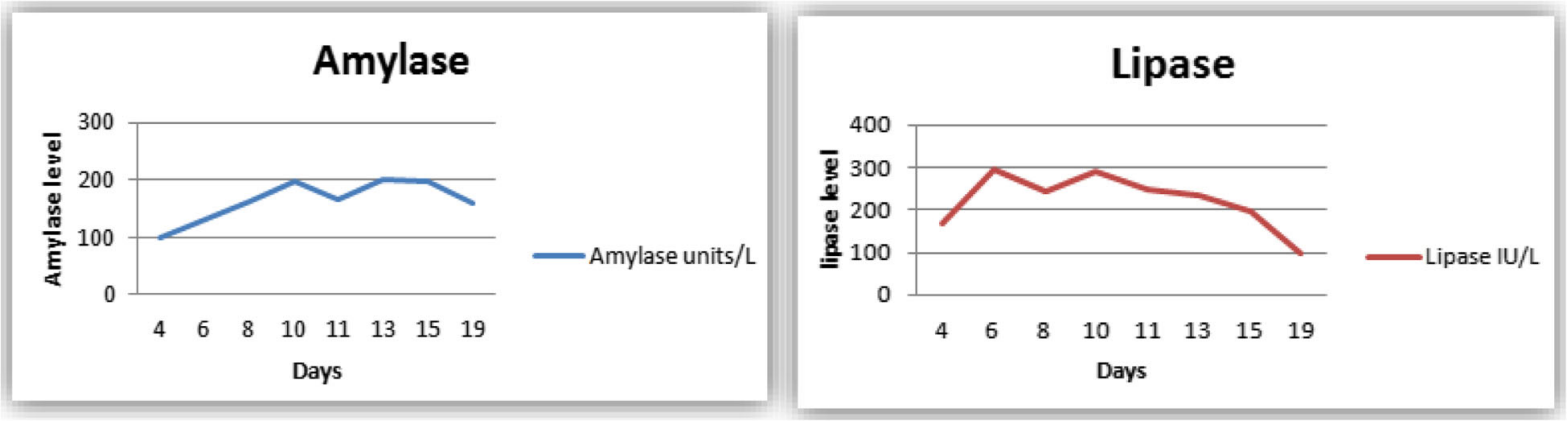
Trend in the pancreatic enzymes amylase and lipase over the course of the patient’s hospital stay

On the **seventh day of his hospital stay**, the patient’s abdominal pain and diarrhea started to improve, and his stool culture was reported to be positive for *Salmonella* group B. Repeated amylase analyses initially showed an upward trend but then trended down (day 15 of admission), and repeated lipase analyses showed a downward trend (day 11 of admission), correlating with clinical improvement (Fig. 3). Repeated inflammatory marker (CRP) analyses showed a downward trend, from 123 mg/L (0–5 mg/L) (on day 6 of admission) to 13 mg/L (0–5 mg/L) (on day 11 of admission).

Thereafter, the patient continued to show gradual improvement in his abdominal pain, fever, and diarrhea, and he was gradually started on a regular diet. His fever subsided on day 11 of the hospital stay, as did his abdominal pain and diarrhea. Later, the patient was discharged home with full recovery and with a close follow-up with gastroenterology, infectious disease, and general pediatric teams. The patient presented to the outpatient clinic for follow-up, and he was completely well with no further concerns. US repeated on day 15 of illness (outpatient setting) showed a complete resolution of the fluid pockets that were seen before.

## Discussion and conclusion

The most common clinical presentation of salmonellosis is acute enteritis, in which the child will present with an abrupt onset of nausea, vomiting, abdominal pain and cramps, located primarily in the periumbilical area and right lower quadrant. This stage is typically followed by mild to severe watery diarrhea and sometimes by diarrheal stool containing blood and mucus. A large proportion of children with acute enteritis are febrile, although younger infants may exhibit a normal or subnormal temperature. *Salmonella* gastroenteritis can be associated with acute dehydration and complications that result from delayed presentation and inadequate treatment [1].

A total of 1–5% of children with *Salmonella* gastroenteritis can develop transient bacteremia. Following bacteremia, salmonellae have the propensity to seed and cause focal supportive infection of many organs. The most common focal infections involve the skeletal system, meninges, intravascular sites, and sites of pre-existing abnormalities [1].

Acute pancreatitis complicating *Salmonella* gastroenteritis is a rare complication among the pediatric population [7]. This complication has been reported in adult patients with typhoid fever and nontyphoid *Salmonella* infection, but there are few reported cases in the pediatric population [7].

Most cases of *Salmonella* gastroenteritis are associated with biochemical pancreatitis that is characterized by an acute elevation of serum amylase and lipase without clinical correlation. However, there is no agreement that acute pancreatitis could be regarded as a complication of *Salmonella* infection [9].

Boyd [10] mentioned that necropsy on some patients who died from *Salmonella* food poisoning showed subtle diffuse acute interstitial pancreatitis. Since these patients died from bacterial or endotoxin shock, pancreatitis might be one of the signs of multiorgan failure. Later, two studies were conducted retrospectively on patients with *Salmonella* enteritis. Renner et al. [11] reported that concomitant pancreatitis was observed in 62% of patients and that the course of pancreatitis was mild to moderate in most of the patients. Murphy et al. [12] found that none of the 51 patients who presented with *Salmonella* infection had clinically apparent pancreatitis, and one patient had a mild elevation of serum amylase without clinical correlation. The interpretation of such inconsistent conclusions from both retrospective studies is not clear.

Hermans et al. [13] studied 14 patients with typhoid fever and found that 7 patients had elevated serum amylase and lipase levels on admission, and 4 out of the 14 patients showed clinical signs of pancreatitis. Baert et al. [14] studied 31 patients with *Salmonella* gastroenteritis and found that 22% of patients experienced an elevation in serum lipase. In each patient, pancreatitis was mild with minimal changes in the US. On the basis of these studies, we concluded that *Salmonella* infection can be associated with chemical pancreatitis but not necessarily with clinically evident pancreatitis.

The mechanism of pancreatitis in patients with *Salmonella* infection is not well understood, although hematogenous or lymphatic spread are the proposed theories. Direct penetration of the pancreas via the migration of salmonellae to the pancreatic duct from the duodenum and biliary tree is another theory, and finally, it is also theorized that toxin-induced or immune-mediated pancreatitis can be associated with *Salmonella* infection [15] [16].

Our patient had multiple reasons for his abdominal pain. Although he tested positive for GAS, the history and physical examination were not supportive of such infection. Therefore, we believe that the positive rapid antigen detection test indicated a carrier state rather than a true infection. We also believe that the pain and the high-grade fever in this patient may have been attributable to intra-abdominal abscesses, as manifested in the US by multiple fluid pockets that responded well to antibiotic therapy. However, this possibility is contradicted by the nature of the pain and its location. Since the pain was mainly located in the epigastric area and aggravated by food intake, the diagnosis of AP rather than another pathology is suggested, but this is not 100% accurate. The fluid pockets were mainly located in the lower abdomen and therefore should have different pain characteristics.

The patient’s clinical response was excellent. Whether the patient’s improvement was due to antibiotics or supportive management for AP is unknown. Thus, whether *Salmonella* can lead to clinically evident pancreatitis remains unclear, and further studies must be done to answer this question. In this case, abdominal CT was the diagnostic imaging method of choice and it is a superior test compared to abdominal US that might have solved the confusion, but unfortunately, it was not done, as the family refused any further imaging.

In conclusion, although AP is a rare complication of *Salmonella* infection in the pediatric population, general pediatricians should entertain, the possibility of AP when patient presents with severe abdominal pain mimicking the characteristics of the pain associated with AP. Early screening and intervention will lead to better outcomes by decreasing the mortality and morbidity rates associated with *Salmonella* infection.

## Abbreviations

AP: Acute pancreatitis
IV: Intravenous
GAS: Group A streptococcus
CRP: C-Reactive protein
US: Ultrasound
CT: Computerized tomography
WBC: White blood cells
INSPPIRE: International Study Group of Pediatric Pancreatitis
